# The Use of Medicinal Plants in the Riverside Community of the Mazagão River in the Brazilian Amazon, Amapá, Brazil: Ethnobotanical and Ethnopharmacological Studies

**DOI:** 10.1155/2019/6087509

**Published:** 2019-04-10

**Authors:** Rosângela do Socorro Ferreira Rodrigues Sarquis, Ícaro Rodrigues Sarquis, Iann Rodrigues Sarquis, Caio Pinho Fernandes, Gabriel Araújo da Silva, Raullyan Borja Lima e Silva, Mário Augusto Gonçalves Jardim, Brenda Lorena Sánchez-Ortíz, José Carlos Tavares Carvalho

**Affiliations:** ^1^Graduate Program in Biodiversity and Biotechnology, Federal University of Amapá (UNIFAP), Brazil; ^2^Laboratory of Pharmaceutical Research, Pharmacy Course, Department of Biological Sciences and Health, Federal University of Amapá (UNIFAP), Rodovia Juscelino Kubitscheck, Km 02, 68902-290 Macapá, AP, Brazil; ^3^Biochemistry Laboratory, Nursing Course, Department of Health Sciences, Faculdade Estácio de Macapá, Brazil; ^4^Phytopharmaceutical Nanobiotechnology Laboratory, Pharmacy Course, Department of Biological and Health Sciences, Federal University of Amapá (UNIFAP), Brazil; ^5^Laboratory of Organic Chemistry and Biochemistry, Collegiate Degree in Chemistry, State University of Amapá (UEAP), Brazil; ^6^Center of Biodiversity, Institute for Scientific and Technological Research of Amapá (IEPA), Brazil; ^7^Paraense Emílio Goeldi Museum (MPEG), Coordination of Botany, Brazil

## Abstract

The inhabitants of the floodplain of the Mazagão River in the State of Amapá in the Brazilian Amazon have inherited from indigenous African and Cabocla cultures indications for the use and forms of preparation of medicinal plants to cure diseases of the body and spirit. This study aimed to perform an ethnopharmacological survey of medicinal plants used by the riparian community of the floodplains of the Mazagão River, in the State of Amapá. In this study, we chose semistructured interviews with socioeconomic, ethnopharmacological, and ethnobotanical aims. The collection of medicinal plants occurred during guided tours. The Use Value (UV), Informant Consensus Factor (ICF), Correction Factor (CF), and Fidelity level (FL) were calculated. There were 130 species of medicinal plants, distributed in 116 genera and 57 families; Fabaceae (16), Lamiaceae (14), Euphorbiaceae (7), and Arecaceae (6) include 33.33% of the total species sampled. All 95 native species of floodplain forests were previously described, and 35 are exotic species. The species with the highest UV (≥ 0.5) at the mouth of the Mazagão River were* Carapa guianensis* (0.91),* Pentachlethra macrolo*ba (0.83),* Dalbergia subcymosa* (0.77),* Uncaria tomentosa* (0.75),* Otacanthus azureus* (0.62),* Virola surinamensis* (0.62),* Hura crepitans* (0.58),* Euterpe oleracea* (0.56), and* Arrabidaea chica* (0.51). These species were also the ones that presented the highest ICF among the informants and 100% in FL for a specific therapeutic use. The study is comprised of 16 categories of therapeutic use, of which the majority of the plants used are related to diseases such as microbial infections (20.67%, 73 species), gastrointestinal disorders (13.31%), and inflammation (11.61%). The results showed that knowledge about the use of medicinal plants along the rivers and streams that form the mouth of the Mazagão River is evenly distributed. Most of the interviewees present diversified knowledge about the medicinal resources because they have a close relationship with the floodplain forest. Native species of this forest predominate among the most commonly used medicinal plants as subsidies for future pharmacological studies.

## 1. Introduction

The floodplain forest is characterized by flood-prone areas influenced by white water rivers with high nutrient-rich sediment loads of Andean or pre-Andean origin, which occupy approximately 2/3 of the flood-prone areas in the Amazon [1]. These forests are inhabited by riparian people, human populations who live on the flood-prone river banks and who produce their food through vegetable extractivism, logging, fishing, handicrafts, and shrimp trapping [2, 3]. The floodplain has always played a central role in the development of the Amazon region, playing different roles in the food and economic survival of riparian dwellers at each period of their history [4, 5].

The riverside communities of the tropical floodplains coexist with a great diversity of natural resources, and they develop some exploration techniques for their own survival, aiming for the establishment of their own management systems that allows them to achieve their needs with low environmental damage, all of this based on their experiences [6–8].

The use of medicinal plants for the treatment of diseases is related to human evolution itself; its use has been reported in all time periods, in all social strata, and for almost all of humanity. The use of medicinal plants for therapeutical purposes in developing countries such as Brazil can be used as an alternative treatment [9, 10]. Phytotherapy and the use of medicinal plants are traditionally part of popular medicine based on the knowledge of different populations, users, and practitioners. It is an effective form of primary health care for the lower income population. Medicinal plants have strongly contributed to the development of new therapeutic strategies through the isolation and identification of its secondary metabolites. These are known to act directly or indirectly through several molecular and cellular targets [11].

The use of medicinal plant-based medicines and the popular knowledge itself implies the need to implement basic research to clarify and confirm information about the actions of the plants, minimizing side, and toxicological effects so that their use is reliable and safe [12].

Historically, the city of Mazagão was founded in the 16th century in North Africa, then it was transferred in the eighteenth century to Portuguese America. It is located on the banks of the Mutuacá River in the State of Amapá, at the Brazilian Amazon rainforest [13]. The New Village of Mazagão, lost in the banks of the Amazon rainforest, was a point of overlapping of cultures, dualities, and conflicts. The memory of the city of the Moroccan coast was diluted and adapted to the new conditions of Portuguese America, building a new urbanity [14]. At that time, the African Mazagans had to live with the outbreak of malaria, a tropical disease endemic to the region; and in order to survive they used traditional knowledge of the use of medicinal plants of the riverside and indigenous communities [15].

Though cultural aspects such as the religious festivals in praise of St. James were preserved and, to this day in July, the battle waged between the Moors and Christians on the coast of Africa is remembered, new practices also emerged from the cultural synthesis of the Mazans, with indigenous people, slaves, and riparians [16, 17]. In the floodplain forest regions of the Eastern Amazon, medicinal plants represent the main form of disease treatment for most of the riparian populations living in this ecosystem, as they live geographically isolated from the urban centers, which causes barriers between them and the public services, especially health service and basic sanitation [18, 19]. These riverside communities have a very peculiar way of interacting with the environment where they live; in most of the houses, septic tanks are still used, and they overflow periodically when the river floods. This water is collected for consumption, several domestic tasks and even for the preparation of the açaí wine (*Euterpe oleracea*), which is the main food consumed in the locality, but it does not undergo adequate treatment to be considered potable, making parasites and stomach diseases a recurring problem in the community [18, 20].

The human populations that settled in floodplain regions started to have a very strong connection with the forest, knowing and exploring it, which allowed a greater contact with the vectors of tropical diseases such as malaria, Chagas disease, and tegumentary leishmaniasis, which are endemic diseases of these regions. To treat and control these diseases, they started to use medicinal plants, a knowledge that is acquired through both experience and verbal cultural transmission, which refers to the past knowledge of one generation to another and plays an important historical role as it facilitates human survival through these generations [19]. Therefore, the riverside inhabitants of the floodplain forest possess important collections of plants that are used for therapeutic purposes as they inherited the use and forms of preparation of medicinal plants for the cure of their diseases of the body and spirit from the indigenous African and Cabocla cultures [19]. However, this knowledge is usually restricted and little studied. Undoubtedly, many plants native to floodplains with therapeutic potential remain unknown and will become extinct because logging is reducing forests and decharacterizing traditional communities in the region.

Few ethnobotanical studies have been performed in the area; for instance, Silva [20] surveyed plants used in Carvão District, Mazagão, AP, where 218 different plant species distributed over 69 families were reported. In the riverside communities of Mazagão Velho, Maracá, and Ajurixi Nascimento [21] it has been reported 73 different plant species distributed over 37 families and their medicinal uses. Overall, these ethnobotanical and ethnopharmacological studies indicate an excellent target to pharmacological researches in the north of Brazil [19, 22, 23].

The State of Amapá has rich plant biodiversity, in addition to a great ethnic and cultural diversity, favoring the accumulation of empirical knowledge about medicinal uses of these plants. Considering the importance to rescue this knowledge from traditional communities and systematize it, this study aimed to perform an ethnobotanical and ethnopharmacological survey of medicinal plants used by riverside communities at the mouth of Mazagão River, in the State of Amapá.

## 2. Materials and Methods

### 2.1. Study Area

**Figure 1:**
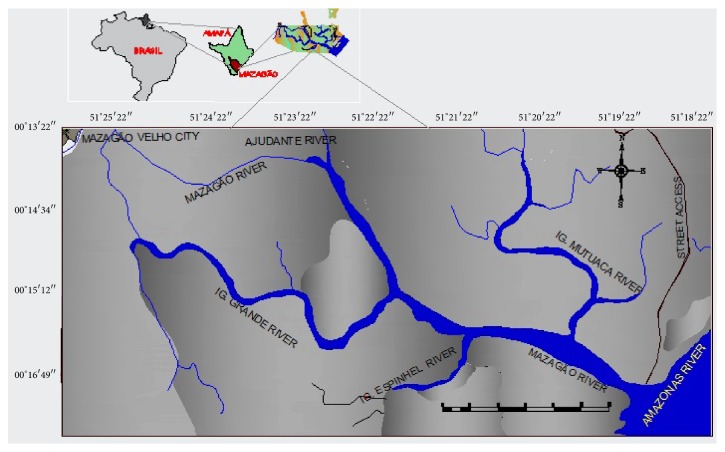
Location map of the mouth of the Mazagão River, Amapá State, Brazilian Amazon.

The municipality of Mazagão occupies an area of 13,131 km^2^, it has a population of 17,032 inhabitants and a population density of 1.30 hab/km^2^ [24], it is 36 km long from the capital Macapá, and it is located at the right margin of the Vila Nova River, south of the State of Amapá under the geographic coordinates 00° 06′ 54′′ S and -51° 17′ 20′′ W (Figure 1). Data collection occurred at the eastern boundary of the municipality of Mazagão in a riparian community that lives at the mouth of the Mazagão River, which has the Mutuacá Mirim, Espinhel, Grande and Ajudante rivers as tributaries. The community at the mouth lives along the Mazagão River, which is situated to the east of the Amazon River and to the west of the Old Mazagão Village, according to Carim et al. [25], the predominant climate type Ami, based on the Köppen classification, with a minimum temperature of 23°C and a maximum temperature of 33°C (annual average of 27°C), with relative humidity above 80%, high rainfall from 2,000 mm/year to 2,500 mm/year, and the Haplic Gleisol soil with a very clayey texture.

### 2.2. Research Authorization

At the beginning of the study, a meeting was organized with the community to present the project and its objectives to obtain community consent for the development of the study. Subsequently, according to the Resolutions of the National Commission for Research Ethics involving Humans and the National Health Council/Ministry of Health, the project was submitted to the Ethics Research Committee of the Faculdade Estácio de Macapá. To do so, the project was registered in the Brazil Platform at http://aplicacao.saude.gov.br/plataformabrasil/login.jsf, in which the following was inserted: interview forms (socioeconomic, ethnobotanical and ethnopharmacological), Community Consent Statement, and the terms of Free and Informed Consent, according to Resolution 466/12 of the National Health Council. This study was approved on 04/13/2016 under opinion number 14.94.994.

### 2.3. Area of Study's Typical Vegetation

Floodplain forests have about 25,000 km^2^ in the area of the Amazonian estuary; it represents about 4.85% from the State of Amapá area and about 15.46% of the coastal estuarine sector [26]. These forests are the flooded ecosystem with the highest biodiversity in the world and have more than 1,000 different species of trees [27, 28]. The local vegetation is cataloged as Alluvial Dense Ombrophilous Forest [29], and the most representative plant families are Fabaceae, Malvaceae, Meliaceae, and Rubiaceae [30]. There is a significant number of palm trees such as* Astrocaryum murumuru* Mart.,* Manicaria saccifera* Gaertn.,* Mauritia flexuosa* L. f.,* Attalea excelsa* (Aubl.) Mart., and* Euterpe oleracea* Mart.; and emerging trees such as* Carapa guianensis *Alblet,* Virola surinamensis *(Rol. ex Rottb.) Warb.,* Mora paraensis *(Ducke) Ducke,* Calycophyllum spruceanum *(Benth.) Hook. f. ex K. Schum.,* Hevea brasiliensis *(Willd. ex A. Juss.) Müll. Arg.,* Cedrela odorata *L., and* Pentaclethra macroloba *(Willd.) Kuntze [30, 31].

The soil under estuarine floodplain forests of Mazagão, AP is, shallow and continuously subjected to flood. In this area, the soil is cataloged as typical Eutrophic Ta Melanic Gleysol [32].

### 2.4. Selection of Informants

In the community, there are 128 houses, all of which were visited, but only the families living in the community for at least 10 years were considered in the interviews. In the selected family, the person who everyone agreed was knowledgeable about home remedies (healer) was the one who was always approached because he/she cultivates and holds knowledge about local plants that are medicinal. A total of 93 forms were applied, representing 72.65% of the total families living in the community.

The study area was investigated to get information from local traditional healers having practical knowledge of medicinal plants; they were interviewed in two villages during April 2016 to May 2018. During the course of the study, six field trips were carried out in the study, and a total 30 days were spent with their local traditional healers. Methods of selecting informants depended upon the distribution of local people having sound knowledge.

All participants agreed to sign the terms of Informed Consent. The interviews were conducted with semistructured forms with open and closed questions about the socioeconomic aspects and the identification of respondents (age, ethnicity, schooling, sex, length of time living in the community, religion, food consumption, family income, and participation in any social project of the government) and information about the medicinal plants used (popular name, part used, indications, and preparation) were recorded. In this work, we considered both plants that are used for diseases well known and treatable by traditional medicine and those indicated for cultural diseases such as “panemeira” (a condition in which the individual is unlucky or has bad luck), bewitching, evil eye, or jinx (the individual is haunted by evil spirits). Other conditions included “ferida brava” (Leishmaniosis ulcer), ringworm (individual with itching in the body), bubo (inflammation in the lymph nodes), “úra” or “fly eye” (fly larvae that lodges in the dermis and causes inflammation in the skin), catarrh in the chest (secretion in the lungs), and “nascida” (a tumor that swells in the skin of any part of the body).

### 2.5. Collection of Botanical Material

The collections were carried out using the guided tour technique [33], which consisted of exploratory walks conducted by the interviewees in the backyard of their residence and in the interior of the forest to identify and collect botanical material from the medicinal plants mentioned during the interview. The referenced species were collected and herborized according to the usual methodology of Fidalgo and Bononi [34]. The plant material was identified by comparison to the Embrapa Eastern Amazon Herbarium; IAN collection; and specialized bibliography [35] and when necessary, via consultations with specialists. Exsiccating of the collected species was included in the IAN of the Embrapa Amazônia Oriental and Herbário Amapaense (HAMAB) of the Institute for Scientific and Technological Research of the State of Amapá. The species mentioned only with the vegetative part were identified, when possible, through specialized literature and using virtual herbarium images for comparison. The spelling and authors of the scientific names were verified in the Missouri Botanical Garden database at www.tropicos.org, and the families were determined according to the classification system proposed by the Angiosperm Phylogenetic Group III [36]. The geographical origin of the species was verified in a specialized bibliography [35, 37].

### 2.6. Data Analysis

The medicinal plants listed by the informants were organized according to the scientific name, popular name, part used, method of preparation, and indications for medicinal use. The reported diseases and symptoms were grouped into 16 categories of therapeutic use according to the indicated body systems. Data were analyzed statistically and described in percentages using Graphpad Prism software (version 6.0). To analyze the relative importance of a species for its ethnomedicinal use, quantitative data (frequency of use and therapeutic indication) were calculated using the Index of Use Value (UV), Loyalty Level (LL), Informant Consensus Factor (ICF), and Correction Factor (CF), according to the mathematical formulas below.

#### 2.6.1. Use Value (UV)

It is a quantitative index that expresses the therapeutic importance of each species. It is calculated by the following formula: UV = Σ^Ui^/n [38], where UV is equivalent to the Use Value of a species, Ui is the number of therapeutic uses reported for each species of plant, and n is the total number of respondents interviewed [39]. The UV parameter helps determine which plant is most frequently used for specific purposes. UV is high when the plant is mentioned by a large number of informants and low when there are few cited uses [40].

#### 2.6.2. Informant Consensus Factor (ICF)

The proposal by Troter and Logan [41] aims to identify the body systems or categories of diseases that have greater relative importance in the site of the study. The ICF is calculated by obtaining the number of citations of uses in each category (N_ur_) minus the number of species used (N_t_), divided by the number of use citations in each category minus 1. The maximum value a category can achieve is 1, which would indicate that there is a well-defined criterion for selecting medicinal plants in the community and/or that use information is shared among the people.

(1) $$\begin{array}{c} ICF=\frac{\left(N_{ur}-Nt\right)}{\left(N_{ur}-1\right)} \end{array}$$

#### 2.6.3. Fidelity Level (FL)

The FL measures the species most frequently used by informants in the study area for a specific therapeutic treatment [42]. FL was calculated using the following formula:

(2) $$\begin{array}{c} FL\left(\;\%\;\right)=\frac{N_{P}}{N}\times 100 \end{array}$$

where Np is the number of citations for therapeutic use given by an informant indicating a species for the highest therapeutic use and N is the total number of informants who cited the species for some use. FL = 100% means that all informants use the species for a therapeutic application, while values below this value mean that the species is used for different purposes [40].

#### 2.6.4. Correction Factor (CF)

The CF determines the difference in the number of informants who cited uses for each species. The CF is calculated by the following formula: CF = N/ICEMC [43], where N is the total number of informants who cited uses for the species and ICEMC is the number of citations of the most frequent species. To extract the importance values related to the species most cited by the interviewees, the following formula was used: Pcusp = LL x CF [44], where Pcusp is the corrected concordance use index, NF is the loyalty level of use, and CF is the correction factor for each species.

## 3. Results and Discussion

### 3.1. Sociocultural Characteristics of Informants

The riverside community at the mouth of the Mazagão River lives on the banks of a floodplain forest; the family income is based on the extractive management of the açaí fruit (*Euterpe oleracea*) and regional shrimp (*Macrobrachium amazonicum*) fishery. In this study, 93 residents were interviewed, ranging in age from 18 to 70 years. The oldest informants with ages ranging from 51 to 70 years were responsible for 51% of citations for use of the medicinal plants; informants ranging from 31 and 50 years old for 38.7% of citations; and informants ranging from 18 to 30 years for 10.3%. When comparing the number of plants cited with the age of the interviewees, it can be seen that the oldest female subjects between 55 and 70 years of age presented the highest number of citations for medicinal plant use. Other ethnobotanical studies also point to the fact of older women knowing more about the medicinal use of plant species [6, 45]. The prevalence is likely higher in older women due to the household duties of caring for the children, the house and the yard, and the place around the house where “girais” (wood artifact, a type of raised bed garden that reaches 3 meters from the ground) are built for the planting of medicinal and food plants. Additionally, the riverside inhabitants live on the edge of the forest, which is periodically flooded, and there is a practice of passing on traditional knowledge about the use of medicinal plants to future generations by word of mouth.

With regard to schooling, 20.4% of the older people were never literate, and those with elementary school education until the 5th grade represent 41% of the community; elementary school through the 8th grade, 20%; high school, 8.6%; incomplete high school, 9%; and higher education, 1%. According to Elisabetsky [46], what makes traditional knowledge of interest to science is the verbal communication of the systematic observation of biological phenomena, made by people who are often illiterate but are certainly insightful regarding the observations of the environment where they live. In this study, it is possible to verify that the older interviewees and those with less education in general are able to recognize a greater number of medicinal plants than are the younger interviewees. This pattern is also observed in other studies, such as those by Hanazaki et al. [47], Pinto [6], and Negrelle et al. [48].

### 3.2. Diversity of Medicinal Plants

A total of 130 medicinal species were identified, which were distributed in 116 genera and 57 families. Fabaceae (16 species), Lamiaceae (14), Euphorbiaceae (7), Arecaceae (6), Asteraceae (4), Rubiaceae (4), Rutaceae (4), Amaranthaceae (4), and Anacardiaceae (4) accounted for the greatest number of species, totaling 48.83% of the sampled species, and 22.5% of the families were represented by only one species. In total, 95 native species of floodplain forest and 35 exotic or introduced species were mentioned (Table 1).

Inventories in Amazonian floodplain forests have shown that Fabaceae has the greatest diversity of species [1, 5, 25, 49, 50]. This fact is demonstrated in this study and is corroborated by Guarado Neto and Moraes [51], who state that when human populations use the native forest for medicinal purposes, the family that has the largest number of species used is the most representative family of the forest.

A study performed by Vásquez et al. [52] at riverside communities of Manacapuru, in the state of Amazonas, reported that 82.7% of the plants used were cultivated, and the family Lamiaceae was the most representative, while in our study the most representative plant family regarding the frequency of citations was Fabaceae. Of the plants cited, 73.07% of them were taken from the forest while 26.93% were cultivated, showing that the people from this community enter the forest to search for the plants.

The “Use” criterion of a species is in the versatility of being mentioned for several therapeutic indications in the community [53, 54]. According to Alexiades [55], the most reliable medicinal uses are those already used by informants, relatives or acquaintances. The species with the highest levels of use agreement and frequency were* Carapa guianensis, Pentaclethra macroloba, Dalbergia monetaria, Uncaria tomentosa, Otacanthus azureus, Virola surinamensis, Hura crepitans *and* Euterpe oleracea*. In other studies, carried out in the Brazilian Amazon, the species* Carapa guianensis, Pentaclethra macroloba, Uncaria tomentosa,* and* Virola surinamensis *were also the most cited in terms of therapeutic use, demonstrating their regional value and the certainty that these plants may become targets in pharmacological research in the region [56, 57].

### 3.3. Used Plant Parts

A total of 170 therapeutic preparations were mentioned, and bark, aerial parts, latex, rhizome, leaf, seed, root, flower, inflorescence, and fruit were the parts of the plant used in the preparations. The most used parts were leaf (40%), bark (32.95%), fruits (7.64%), root (4.7%), and inflorescence (4.7%). In the community, parts of the same plant may be used for distinct indications, such as in* Carapa guianensis, Pentaclethra macroloba, *and* Virola surinamensis, *whose oil extracted from the seed is used topically in the case of inflammatory processes of the skin, as a repellent and for the healing of wounds, and the leaves and barks are used in oral preparations by decoction for inflammation of the digestive, urinary, and reproductive systems.

The leaves are vegetative structures that stand out in the methods of ethnomedicinal preparations by decoction, maceration, and infusion in traditional communities in Brazil [50, 58, 59] and in other regions of the world [39, 60–63]. One of the reasons is the ease of collection [64] and the production of secondary metabolites present mainly in the leaves [65]. Phytochemical studies with leaves contained flavonoids, tannins, saponins, steroids, and triterpenoids [66, 67]. According to Matos and Matos [68], flavonoids have direct action on capillaries and potentiate ascorbic acid, whose hemorrhagic action and anti-inflammatory action are similar to cortisone. Tannins are used in the treatment of burns, in the recomposition of exposed tissue proteins and in the formation of slightly antiseptic coating [66]. Steroids have important therapeutic properties (cardiotonic, anabolic, contraceptive, and anti-inflammatory) [69]. According to Robbers et al. [70], the triterpenoids have antimicrobial and antitumor action, but some are very toxic to the human body.

### 3.4. Forms of Ethnomedicinal Preparations

The preparation forms were classified as decoction, infusion, syrup, maceration, oil, hydration water, latex, tincture, and poultice (Table 1). Decoction was the most used form of preparation (59.4%), followed by maceration (11.5%) and infusion (9.7%), and the other six forms of preparation combined corresponded to 19.4%. According to Amorozo and Gély [71] and Lisboa [72], decoction is the most common way of administration home remedies in Amazonian communities. Decoction is performed so that the home remedy is stored in a refrigerator and has more use lifetime. Teas obtained by decoction or infusion are consumed orally and can be used in baths for various types of diseases, including the cultural diseases reported in this work such as “nascida”, “panemeira”, bewitching, evil eye, “úra”, and bubo.

In the community, women collect the fruits of* Carapa guianensis *(andiroba) and* Pentaclethra macroloba *(pracaxi), which are dispersed in the water, for the production of medicinal oil, a common management practice to commercialized them in the city of Macapá.

Maceration is a preparation that uses barks, leaves, branches and roots that are immersed in water and/or alcohol, are indicated for inflammation, diarrhea and other intestinal disorders, and are consumed by mouth. In the community, the latex that flows from the trunk of medicinal tree species is applied to wounds, and in this study, it is also collected in medicinal bottles for oral use. According to Viega and Scudeller [73], the use of bark, root, and latex in the preparation of home remedies is known as medicinal bottle, and it is a preparation that is widely disseminated throughout the Amazon and other areas of the world.

### 3.5. Therapeutic Indications

The riverside residents reported 2103 phytotherapeutic uses for medicinal plants, which were grouped into 16 categories of therapeutic use. For this, the type of body system involved was related to the disease and symptoms, as well as the medicinal species used in the community. It was observed that most of the plants used are related to diseases such as microbial infections (20.67%, 73 species), gastrointestinal disorders (13.31%, 47 species) and inflammations (11.61%, 41 species), emphasizing that 1 specie can be cited for several diseases (Table 2). Studies carried out in traditional communities in Brazil often point to several plant species for problems of the gastrointestinal system, including parasitic diseases and infections caused by microbial agents [57, 74, 75]. These results corroborate studies carried out in communities that do not have basic sanitation in developing countries alongside Latin America [38, 57, 76–79], and this ranking may therefore be related to economic conditions and regional habits as the riparian region studied does not present basic sanitation, and the community uses septic tanks and collects water directly from the river for their essential needs.

Leão et al. [80] performed an ethnobotanical survey in Santa Barbara, state of Pará. Similarly to this study, the authors observed that the major diseases and symptoms treated by the community were gastrointestinal disorders, microbial infections, and inflammations. This was also reported in other study by Amorozo [58].

**Figure 2:**
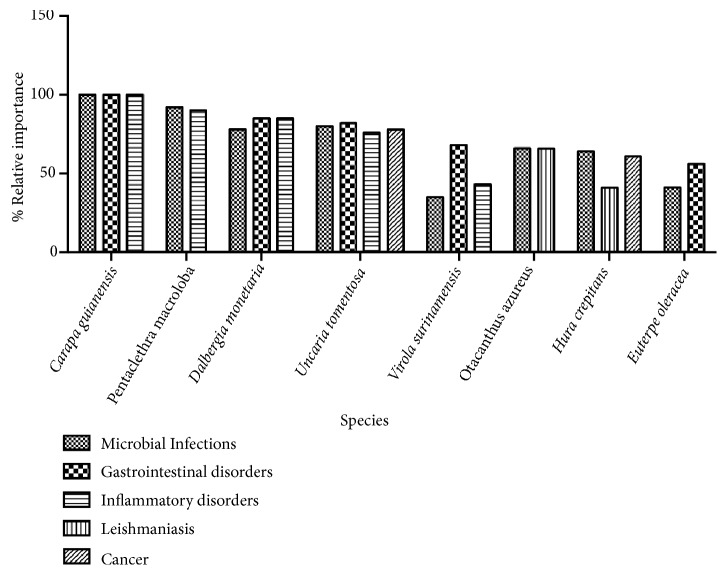
Relative importance of the most cited species in relation to main categories of therapeutic use in the community.

In this study, the species* Carapa guianensis* (100%),* Pentaclethra macroloba* (90-92%),* Dalbergia monetaria *(78-85%),* Uncaria tomentosa* (80-82%),* Virola surinamensis* (43-68%),* Otacanthus azureus* (66%),* Hura crepitans* (41-64%), and* Euterpe oleracea* (41-56%) (Figure 2) are widely used by the riverside community to treat microbial infections, gastrointestinal disorders, inflammatory conditions, leishmaniasis, and cancer, as seen by the use agreement index (Table 3). According to Vendruscolo and Mentz [53], this index indicates the most promising species to perform pharmacological researches according to their use.

Ethnopharmacological studies performed with the species* C. guianensis, P. macroloba*,* D. monetaria,* and* U. tomentosa* show that species are popularly used to treat abscesses, asthma, skin diseases, infectious diseases, deep wounds, gastritis, inflammations, gastric ulcer [16, 23, 44, 80]. Currently, this is being confirmed experimentally in studies with the oil from the seeds of* C. guianensis* that show its anti-inflammatory activity [35, 80], antiallergic activity [81–83], and wound-healing activity [84, 85]. Also, phytochemical studies attribute anti-inflammatory and antiallergic activity to the tetranortriterpenoids, main molecules of* C. guianensis* oil [86]. The same occurs with* U. tomentosa*, whose extract is reported to have antimicrobial, anti-inflammatory and anticancer activity* in vitro* and* in vivo* [87]; this is due to the presence of alkaloids, triterpenic heterosides, and polyphenols, mainly tannins [88–90].

The species* P. macroloba* has some medicinal application against snakebites. Triterpenic saponins isolated from its fruit were reported to be effective against snake venom [91]. Also, the essential oil of* O. azureus* has bactericidal, antioxidant [92], antifungal [93], and leishmanicidal activity [94]. However, the species* D. monetaria, V. surinamensis, O. azureus*,* H. crepitans, *and* E. oleracea* still need further* in vitro* and* in vivo* pharmacological research to corroborate their use in folk medicine.

Of the informants, 100% believe in the efficacy of medicinal plants in common diseases such as fever, diarrhea, and infections and prefer to use them because they understand that there are no side effects and because it is a therapeutic resource free of cost and easily obtainable in the community; however, when serious complications result from malaria and heart disease, they prefer drugs from the pharmacy because they works faster.

### 3.6. Comparison of the Different Indices

In this study, UVs between 0.91 and 0.56 were from native medicinal plants that are frequently used as an ethnomedicinal resource by the riparians:* Carapa guianensis* (0.91),* Pentachletra macroloba* (0.83),* Dalbergia monetaria* (0.77),* Uncaria tomentosa* (0.75),* Otacanthus azureus *(0.62),* Virola surinamensis* (0.62),* Hura crepitans* (0.58), and* Euterpe oleracea* (0.56). These species were the most versatile in the therapeutic preparations and obtained a use index ranging from 100% to 56%; they also were the most frequent in the study area, with a loyalty index of 100% for a specific therapeutic use (Table 1). The most important species for a community are those that have the highest Use Value, and they should be prioritized for conservation [95].

The species* Carapa guianensis* and* Uncaria tomentosa* have been among the most phytochemically studied species in recent years. Studies show that* C. guianensis* (andiroba) oil has anti-inflammatory [35, 95], antiparasitic [96], antispasmodic [97], repellent, antiallergic, antirheumatic and healing properties [98]. Pharmacological tests performed* in vitro *and* in vivo *with* U. tomentosa *(jupindá) showed antioxidant [99], anticancer [100], anti-inflammatory [101], antimicrobial [102], antiherpetic [103] and antidiabetic [104] activities. In turn,* Pentaclethra macroloba, Virola surinamensis*,* Dalbergia monetaria*,* Otacanthus azureus*,* Virola surinamensis*,* Hura crepitans,* and* Euterpe oleracea* are little known pharmacologically.

 Eight floodland forest native species are mentioned for the first time in an ethnobotanical study in the region, and no pharmacological studies have been found for* Allamanda cathartica* (0.01), which is used for the treatment of intestinal parasites*; Astrocaryum murumuru *(0.01), used for the treatment of eye infections of dogs and skin irritations of other animals;* Calophyllum brasiliense* (0.01), used for joint inflammations and skin ulcers;* Passiflora tholozanii* (0.02), used for leishmaniasis ulcers, cancer, depression and soothing;* Manicaria saccifera* (0.04), used for gastritis;* Pourouma guianensis* (0.04), used for leishmaniasis joint inflammation and ulcers;* Triplaris surinamensis* (0.04), used for the treatment of joint inflammation; and* Unonopsis floribunda* (0.04), used for joint and stomach inflammation. Although many species are reported for the treatment of the diseases mentioned, these species deserve attention because the region is going through a rural exodus, and people with this knowledge are decreasing in number and have no successors of that knowledge, which is traditionally passed by word of mouth.

The Informant Consensus Factor (ICF) was calculated for 16 categories of therapeutic uses (Table 2). Leishmaniasis is endemic in the riverside area and recorded an ICF value of 0.9, followed by cancer (0.93), gastrointestinal disorders such as diarrhea, vomiting, and gastritis (0.89), inflammation in the uterus and burns (0.88), diabetes and albumin (0.87), and microbial, respiratory infections and pain, fever and cold symptoms (0.86); these include diseases such as itching, uterine infections, body aches, insect bites, flu, catarrh in the chest and cough. Nervous system disorders such as epilepsy, convulsions and depression had ICF values of 0.77; malaria, 0.73; renal and bile calculi, 0.72; infarction, bleeding, and high blood pressure, 0.71; followed by verminoses, 0.66; gynecological problems after childbirth, 0.64; and joint inflammation, 0.54.

High ICF values clearly showed that the community uses medicinal plants for their health problems and that there are well-defined choice criteria, which are shared orally [48, 105]. A total of 73 medicinal plants were used for microbial infections, followed by 47 that are referred for the gastrointestinal system and 41 for general inflammation in the body. According to the interviewees, the therapeutic use of plants in the community is the first alternative for the treatment of health problems, and in many cases, it constitutes the only immediate resource for this purpose, as there are difficulties in accessing allopathic medicines because they live far from urban centers, have small boats as transportation means, which are called “rabetas”, and do not have public health units for simple clinical care for diarrhea, headaches and infections. According to Lima et al. [106], the rich traditional knowledge of communities living in isolation in the Amazon arises from the need to have an alternative therapeutic treatment, caused by the limited access to the public health network and the great cultural influence of these peoples.

In the study, the floodland forest native species with the highest values of relative importance, for the treatment of the most frequent diseases in the community, are as follows (Table 3):* Carapa guianensis, Hura crepitans, Otacanthus azureus Uncaria tomentosa, Uncaria guianensis, Pentaclethra macroloba, Copaifera sp., Dalbergia monetaria, Spondias mombin, Virola surinamensis, Symphonia globulifera*,* Parkia pendula*,* Maquira coriacea, Croton urucurana, Quassia amara, Genipa americana, Vismia macrophylla, Gustavia augusta, *and* Passiflora tholozanii. *They are the most versatile because they can be used in various therapeutic treatments.

## 4. Conclusion

This is a pioneer study of the riparian community in flooded areas of Mazagão, and it shows that knowledge about plants and their medicinal uses is diverse and widespread in the community, likely because of the high incidence of tropical diseases such as malaria and leishmaniasis as well as the difficulty in accessing medicines distributed by the government and easy access to local plants. The riparians listed 130 ethnospecies, of which 95 are mostly native trees of lowland forest. In the study, the residents showed that they use conservation practices such as not annealing the individuals when collecting bark/bast; in addition, after extracting medicinal oil from the copaiba trunk, they always allow one year for the plant to recover, and if needed, they search for another trunk so as to not to exhaust the resource. They also collect* Carapa guianensis* (andiroba) and* Pentachletra macroloba* (pracaxi) fruits, which are dispersed in the water, for extraction of the medicinal oil, which is marketed in urban areas. Residents report that they have a potential consumer market for these medicinal oils and that organization is lacking to make the production of this oil profitable. Therefore, native medicinal species should be prioritized for conservation, as riparians depend on the collection of these plants as the main drugs for the region's endemic and cultural diseases; furthermore, these plants may be used for future pharmacological studies. Studies of this nature reinforce the importance of the relationship between the community and biodiversity, valuing, and bringing visibility to the ethnobotanical and ethnopharmacological knowledge they possess. The systematization of knowledge about these resources rescues popular knowledge, contributing not only to the conservation of diversity but also to the preservation of a rich and important cultural heritage.

## Figures and Tables

**Table 1:** Vegetal species used as medicinal plants by residents at the mouth of the Mazagão River, State of Amapá, Brazilian Amazonia, and the use value.

Scientific Name (Family) Voucher n°	Local name	Distribution	Plant part used	Traditional uses	Preparation method and use form	UV
(1) ***Acmella oleracea* (L.) R.K. Jansen** (Asteraceae), IAN 195948	Jambú	Native	Leaves and inflorescences	Pain, inflammation, Anemia and malaria.	Decoction; 1 cup 3x a day for 7 days	0,09

(2) ***Acrocomia aculeata* (Jacq.) Lodd. ex Mart.** (Arecaceae), IAN 195943	Mucajá	Native	Root and leaves	Catarrh in the chest (secretion in the lung) and cough.	Decoction; 1 spoon 4 x a day for 7 days.	0,02

(3) ***Adenocalymna alliaceum *Miers** (Bignoniaceae), IAN 196009	Cipó d'alho	Native	Leaves and bark	Cold, inflammation of the throat and fever.	Decoction; 1 scoop 2 x daily and inhale 2x steam for 7 days.	0,17

(4) ***Aeollanthus suaveolens *Matt. ex Spreng.** (Lamiaceae), IAN 195989	Caatinga de mulata	Introduced	Leaves	Irritation of the nerves and strong headache.	Dyeing and infusion; bathe the body 2 x in the day for 10 days.	0,46

(5) ***Allamanda cathartica *L.** (Apocynaceae), IAN 196010	Buiuçú	Native	Exsudate and leaves	Elimination of lice and antihemintics.	Decoction; 1 spoon 2 x a day for 7 days and soak hair 1 x a day for 10 minutes for 3 days.	0,01

(6) ***Allium sativum *L.** (Liliaceae), HAMAB 8938	Alho	Introduced	Leaves	Flu, pain in the stomach and antihemintics	Decoction; 1 cup 3 x daily for 7 days.	0,03

(7) ***Aloe vera *(L.) Burm.** (Asphodelaceae), IAN 194110	Babosa	Native	Leaves	Healing.	Cataplasm; spend 3 x a day until the wound is dry.	0,10

(8) ***Alstroemeria brasiliensis *Spreng.** (Alstroemeriaceae), IAN 196011	Carajurú, cajuru	Native	Bark and root	Malaria.	Decoction; 1 cup 3 x daily for 7 days.	0,01

(9) ***Alternanthera brasiliana *(L.) Kuntze** (Amarathaceae), IAN 194099	Perpétua do mato, sempre-viva.	Native	Leaves	Elimination of kidney stone, antitumor, infections of the liver and bladder.	Decoction; leaves of *Alternanthera brasiliana*, *Alternanthera dentata* and *Alternanthera ficoidea*, ingest 1 cup 4 x a day for 20 days.	0,20

(10) ***Alternanthera dentata *(Moench) Stuchlík ex R.E. Fr.** (Amaranthaceae), IAN 195934	Cibalena	Native	Leaves	Elimination of kidney stone, infections of the liver, stomach pains.	Decoction; leaves of *Alternanthera brasiliana*, *Alternanthera dentata* and *Alternanthera ficoidea*, ingest 1 cup 4 x a day for 20 days.	0,01

(11) ***Alternanthera ficoidea* (L.) Sm.** (Amaranthaceae), IAN 195952	Piriquitinho verde	Native	Leaves	Elimination of kidney stone, inflammation of the uterus and ovary.	Decoction; leaves of *Alternanthera brasiliana*, *Alternanthera dentata* and *Alternanthera ficoidea*, ingest 1 cup 4 x a day for 20 days.	0,07

(12) ***Anacardium occidentale *L.** (Anacardiaceae), IAN 195977	Caju	Native	Leaves	Diarrhea, uterine cramps.	Decoction; 1 cup 3 x daily for 7 days.	0,16

(13) ***Annona muricata *L.** (Annonaceae), IAN 195945	Graviola	Native	Leaves	Diarrhea and skin sore.	Decoction and infusion; 1 cup 3 x daily for 7 days.	0,03

(14) ***Arrabidaea chica* (Bonpl.) B. Verl.** (Bignoniaceae), IAN 194107	Pariri	Native	Leaves	Anemia, diarrhea, washing of wounds, yellow skin and swollen body of pregnant woman.	Decoction and maceration; 1 cup 3 x daily for 7 days.	0,51

(15) ***Astrocaryum murumuru *Mart.** (Arecaceae), IAN 196007	Murumuru	Native	Fruit	Dog eye inflammation and irritation in human eyes.	Decoction and maceration; 1 cup 3 x daily for 7 days.	0,01

(16) ***Bactris maraja *Mart.** (Arecaceae), IAN 196006	Marajá	Native	Root	Diarrheal, inflammation of the uterus.	Decoction and maceration; 1 cup 3 x daily for 7 days.	0,02

(17) ***Bauhinia splendens *Kunth.** (Fabaceae), IAN 196005	Escada de jabuti	Native	Bark	Joint pain, rheumatism and syphilis.	Decoction and maceration; 1 cup 3 x daily for 7 days.	0,03

(18) ***Bixa orellana *L.** (Bixaceae), IAN 196001	Urucum	Native	Fruit	Repellent, burn, diarrhea and asthma.	Syrup and decoction; 1 cup 3 x daily for 7 days. Mix with *Carapa guianensis* Aubl. oil and pass on the body 1 x a day.	0,08

(19) ***Brassica oleracea *L.** (Brassicaceae), IAN 195942	Couve manteiga	Introduced	Leaves	Anemia and cholesterol.	Decoction; 1 cup 2 x daily for 7 days.	0,03

(20) ***Bryophyllum pinnatum *(Lam.) Oken** (Crassulaceae), IAN 195954	Pirarucu	Introduced	Leaves	Irritation of the skin.	Decoction; pass on the skin 2 x in the day for 7 days.	0,03

(21) ***Libidibia ferrea *(Mart. ex Til.) L.P. Queiroz** (Fabaceae), IAN 195940	Jucá	Native	Fruit	Bleeding, inflammation in general, washing of wounds and swelling of the legs.	Decoction and maceration; 1 cup 3 x daily for 7 days.	0,01

(22) ***Caladium humboldtii *Schott** (Araceae), IAN 194115	Brasileirinho	Native	Leaves	Fever, pain in the uterus and inflammation in general.	Decoction; 1 cup 3 x daily for 7 days.	0,07

(23) ***Calophyllum brasiliense* Cambess.** (Clusiaceae), IAN 195937	Jacareúba	Native	Exsudate and Bark	Joint pain, rheumatism and skin sore.	Exudate; pass on the skin 4 x a day for 7 days.	0,01

(24) ***Calycophyllum spruceanum* (Benth.) Hook. f. ex K. Schum. **(Rubiaceae), IAN 195980	pau mulato	Native	Bark	Itching, inflammation in general and insect stings.	Decoction; 1 cup 3 x daily for 7 days.	0,04

(25) ***Carapa guianensis *Aubl.** (Meliaceae), IAN 194102	Andiroba	Native	Seed	Inflammation in general, massage the pregnant belly to put the baby in place.	Oil; ingest 1 teaspoon 4 x daily for 10 days, topical use 2 x daily for 7 days.	0,91

(26) ***Cecropia pachystachya *Trécul** (Urticaceae), IAN 195956	Embaúba vermelha	Native	Leaves	Skin irritation and verminoses.	Decoction; 1 teaspoon 4x a day for 7 days.	0,08

(27) ***Cedrela odorata *L.** (Meliaceae), IAN 195971	Cedro	Native	Bark	Verminose, skin irritation, intestinal infections.	Decoction and maceration; 1 cup 4 x daily for 7 days.	0,08

(28) ***Cereus jamacaru* DC.** (Cactaceae), IAN 195976	Mandacarú.	Native	Branches and root	Pulmonary problems, skin infections, sores and kidney stones.	Decoction; 1 teaspoon 4x a day for 10 days.	0,01

(29) ***Chenopodium ambrosioides *L.** (Amaranthaceae), IAN 195978	Mastruz	Introduced	Leaves	Pain, inflammation in general and rheumatism.	Decoction; 1 teaspoon 4x a day for 7 days.	0,11

(30) ***Cichorium intybus *L.** (Asteraceae), IAN 195979	Chicória	Introduced	Leaves and root	Inflammation in general, pain and fever.	Decoction; 1 cup 2 x daily for 7 days.	0,10

(31) ***Cinnamomum zeylanicum *Blume.** (Lauraceae), IAN 195982	Canela	Introduced	Leaves and bark	Flu, diarrhea, inflammation of the uterus and wounds in the mouth.	Syrup and infusion; 1 cup 2 x daily for 7 days.	0,02

(32) ***Cissus verticillata *(L.) Nicolson & C.E. Jarvis **(Vitaceae), IAN 195983	Cipó-urumucá, cipó-pucá	Native	Leaves	High blood pressure, stroke, anemia, tremors and epileptic seizures.	Decoction; 1 cup 2 x daily for 7 days.	0,03

(33) ***Citrus aurantium *L.** (Rutaceae), IAN 195960	Laranja da terra	Introduced	Bark	Swelling of a pregnant woman, inflammation in general, and itching of the foot.	Maceration; 1 cup 2 x a day at dawn and dusk for 7 days.	0,07

(34) ***Citrus limon *(L.) Burm.** (Rutaceae), IAN 195988	Limão galego	Introduced	Fruit	Rheumatism and inflammation of the kidneys.	Decoction; 1 cup 2 x daily for 7 days.	0,02

(35) ***Citrus limonum *L.** (Rutaceae), IAN 195989	Limão	Introduced	Fruit	Rheumatism and inflammation of the kidneys.	Syrup and infusion; 1 cup 3 x daily and inhale the steam 2 x daily for 7 days.	0,29

(36) ***Cocos nucifera *L.** (Arecaceae), IAN 195990	Côco	Native	Fruit and root	Hydration of child and verminoses.	Decoction of the root 1 cup 2 x a day for 7 days and fruit water will during the day for 7 days.	0,02

(37) ***Copaifera *sp.** (Fabaceae), IAN 195991	Copaiba	Native	Bark and oil	Inflammation of the uterus and ovary, and diseases of the stomach.	Decoction; 1 cup 2 x daily for 7 days.	0,02

(38) ***Costus spicatus *(Jacq.) Sw.** (Costaceae), IAN 195993	Canafiche, canarana	Native	Aerial part	Inflammation of the uterus and ovary, vaginal discharge and syphilis.	Decoction; 1 cup 3 x daily for 7 days.	0,03

(39) ***Crescentia cujete* var*. puberula* Bureau & K. Schum. **(Bignoniaceae), IAN 195959	Cuieiro	Native	Leaves and bark	Malaria, fever and rheumatism.	Decoction; 1 cup 3 x daily for 7 days.	0,05

(40) ***Croton cajucara *Benth.** (Euphorbiaceae), IAN 195994	Sacaca	Native	Leaves and bark	Diarrhea, diabetes, inflammation of the liver, kidneys and bladder.	Decoction; 1 cup 2 x daily for 7 days.	0,01

(41) ***Croton urucurana *Baill.** (Euphorbiaceae), IAN 195996	Forsangue	Native	Bark	Hemorrhagic and inflammation of the uterus.	Decoction; 1 cup 3 x daily for 7 days.	0,18

(42) ***Cucumis anguria *L.** (Cucurbitaceae), IAN 195998	Maxixe	Introduced	Fruit	Eliminate kidney stones, hemorrhoids, inflammation of the kidneys, vomiting.	Decoction; 1 cup 3 x daily for 10 days.	0,01

(43) ***Cymbopogon citratus *(DC.) Stapf** (Poaceae), IAN 195953	Capim marinho	Introduced	Leaves	Inflammation of the uterus and ovary and abdominal pain.	Decoction; 1 cup 3 x daily for 7 days.	0,10

(44) ***Dalbergia monetaria *L. f.** (Fabaceae), IAN 195964	Verônica	Native	Bark	Inflammation, diarrhea and intestinal infection.	Decoction and maceration ingest 1 cup 3 x daily for 7 days.	0,77

(45) ***Dimorphandra gardneriana *Tul.** (Fabaceae), IAN 195995	Faveira	Native	Fruit	High pressure.	Decoction; 1 cup 3 x daily for 7 days.	0,02

(46) ***Eleutherine bulbosa* (Mill.) Urb.** (Iridaceae), IAN 195997	Marupá, Marupázinho	Introduced	Rhizome	Verminoses, wound healing on the skin.	Decoction; 1 cup 3 x daily for 7 days.	0,18

(47) ***Erythrina fusca *Lour.** (Fabaceae), IAN 194108	Assacurana	Native	Bark	Pain in body and head, inflammation and verminoses.	Decoction; 1 cup 3 x daily for 7 days.	0,04

(48) ***Eucalyptus *sp.** (Myrtaceae), IAN 196000	Eucalipto	Introduced	Leaves	Inflammation in general.	Decoction; 1 cup 2 x daily for 7 days.	0,04

(49) ***Eupatorium triplinerve *Vahl.** (Asteraceae), IAN 195946	Japana	Introduced	Aerial parts	Flu	Syrup and infusion; ingest 1 cup 3 x daily for 7 days.	0,09

(50) ***Euterpe oleracea *Mart.** (Arecaceae), HAMAB 10919	Açaí	Native	Bark	Diarrhea and intestinal inflammation.	Decoction; 1 cup 3 x daily for 7 days.	0,56

(51) ***Genipa americana *L.** (Rubiaceae), IAN 196001	Desinflama	Native	Leaves and bark	Diarrhea, sores on the skin, pains and phlegm in the lung.	Decoction; 1 cup 3 x daily for 7 days.	0,03

(52) ***Gossypium hirsutum *L.** (Malvaceae), IAN 195690	Algodão	Introduced	Leaves	Fever of infections.	Decoction; 1 cup 3 x daily for 7 days.	0,21

(53) ***Gustavia augusta *L.** (Lecythidaceae), IAN 196003	Geniparana	Native	Bark, leaves and flowers	Brave wound (leishmaniasis).	Poultry, decoction and maceration, ingest 1 x 2 x daily for 10 days. Topical use 2 x in day for 7 days.	0,04

(54) ***Hevea brasiliensis* (Willd. ex A. Juss.) Müll. Arg. **(Euphorbiaceae), IAN 196068	Seringueira	Native	Exsudate	Brave wound (leishmaniasis).	Exudate as poultice, topical use 2 x in day for 7 days.	0,03

(55) ***Himatanthus sucuuba *(Spruce ex Müll. Arg.) Woodson** (Apocynaceae), IAN 194107	Sucuúba	Native	Bark and exsudate	Verminoses, inflammation and pain in the uterus, malaria and rheumatism.	Cataplasm; topical use 2 x in day for 7 days.	0,04

(56) ***Hura crepitans *L.** (Euphorbiaceae), IAN 195981	Assacú	Native	Bark	Cancer.	Decoction; 1 cup 2 x daily for 10 days.	0,58

(57) ***Hymenaea oblongifolia* Huber** (Fabaceae), IAN 196009	Jatobá da várzea	Native	Bark and fruit	Diarrhea, pain and inflammation in the body.	Decoction and maceration; 1 cup 2 x daily for 7 days.	0,17

(58) ***Hypericum perforatum *L.** (Hyperaceae), IAN 194100	Dipirona, hipericão, erva de são João	Introduced	Leaves and casca	Pain, depression, nervous irritation.	Decoction; 1 cup 3 x daily for 10 days.	0,04

(59) ***Scutellaria agrestis* A. St. Hil. ex Benth.** (Lamiaceae), IAN 195992	Trevo rocho	Native	Leaves and inflorescences	Headache, itchy skin.	Infusion and poultice; topical use 3 x daily for 7 days.	0,01

(60) ***Jatropha curcas *L.** (Euphorbiaceae), IAN 195944	Pião branco	Introduced	Leaves, seed and bark	Headaches, catarrh in the chest, flu, stroke and scarring.	Dyeing and infusion; 1 cup 2 x daily for 10 days.	0,38

(61) ***Jatropha gossypiifolia *L.** (Euphorbiaceae), IAN 195949	Pião rocho	Introduced	Leaves	Broken and evil eye.	Infusion; 1 cup 2 x daily for 7 days.	0,08

(62) ***Centratherum punctatum* Cass.** (Asteraceae), IAN 195987	Anador, melhoral	Native	Leaves	Bleeding and inflammation of the kidneys.	Decoction; 1 cup 2 x daily for 10 days.	0,21

(63) ***Justicia pectoralis* Jacq.** (Acanthaceae), IAN 194100	Anador, melhoral	Native	Leaves	Headache and joint pain.	Decoction; 1 cup 2 x daily for 7 days.	0,21

(64) ***Lecythis pisonis *Cambess.** (Lecytidaceae), IAN 196010	Sapucaia	Native	Bark, leaves and flowers	Diarrhea and inflammation in general.	Decoction; 1 cup 2 x daily for 10 days.	0,01

(65) ***Licania macrophylla *Benth.** (Chrysobalanaceae), IAN 194104	Anoerá ou anuerá	Native	Bark	Diarrhea and bleeding.	Decoction and maceration; 1 cup 3 x daily for 7 days.	0,20

(66) ***Lippiaalba* (Mill.) N.E. Br. ex Britton & P. Wilson** (Verbenaceae), IAN 194109	Camelitana, erva cidreira	Native	Leaves and flowers	Pain and catarrh in the chest.	Decoction; 1 cup 3 x daily for 7 days.	0,01

(67) ***Luffa operculata *(L.) Cogn.** (Cucurbitaceae), IAN 196013	Buchinha ou cabacinha	Native	Leaves, bark and fruit	Flu, abortion and verminosis.	Decoction; 1 cup 3 x daily for 7 days.	0,02

(68) ***Machaerium lunatum *(L.) Ducke** (Fabaceae), IAN 195973	Aturiá	Native	Leaves	Rheumatism.	Decoction; 1 cup 3 x daily for 7 days.	0,03

(69) ***Macrolobium acaciifolium *(Benth.) Benth.** (Fabaceae), IAN 196011	Faveira-araparí	Native	Bark	Healing.	Maceration; 1 x 3 x daily and topical use 2 x daily for 7 days.	0,04

(70) ***Mangifera indica *L.** (Anacardiaceae), IAN 195965	Manga	Introduced	Leaves and bark	Diarrhea and intestinal infections.	Decoction; 1 cup 3 x daily for 7 days.	0,47

(71) ***Manicaria saccifera *Gaerth.** (Arecaceae), IAN 195984	Bussú	Native	Fruit	Inflammation in the stomach and born in the body (furunculosis).	Maceration; 1 cup 3 x daily for 10 days.	0,04

(72) ***Maquira calophylla *(Poepp. & Endl.) C.C. Berg** (Moraceae), IAN 196012	Muiratingarana	Native	Bark	Cancer.	Decoction; 1 cup 3 x daily for 20 days.	0,04

(73) ***Maquira coriacea *(H. Karst) C.C. Berg **(Moraceae), IAN 196013	Muiratinga	Native	Bark	Malaria.	Decoction; 1 cup 3 x daily for 7 days.	0,04

(74) ***Melissa officinalis *L.** (Lamiaceae), IAN 195947	Cidreira, erva-cidreira	Introduced	Leaves and inflorescences	Headaches, digestive problems, anxiety and nervousness.	Infusion; 1 cup 3 x daily for 7 days.	0,03

(75) ***Mentha × piperita* L.** (Lamiaceae), IAN 196014	Hortelânzinho	Introduced	Leaves	Verminoses and inflammations of the kidney, bladder and liver.	Infusion; 1 cup 3 x daily for 7 days.	0,26

(76) ***Mentha arvensis *L.** (Lamiaceae), IAN 196015	Vick	Introduced	Leaves	Eliminate phlegm from the chest, headache and flu.	Syrup and infusion; 1 cup 3 x daily and inhale the steam 2 x daily for 7 days.	0,08

(77) ***Mentha pulegium* L.** (Lamiaceae), IAN 196016	Hortelân grande	Introduced	Leaves	Flu and inflammation of the kidney.	Syrup and infusion; 1 cup 3 x daily and inhale the steam 2 x daily for 7 days.	0,04

(78) ***Mikania hirsutissima DC.*** (Asteraceae), IAN 195691	Cipó-Sucuriju	Native	Bark	Diarrhea and intestinal infections.	Decoction; 1 cup 3 x daily for 7 days.	0,03

(79) ***Minquartia guianensis* Aubl.** (Olacaceae), HAMAB 6910	Acapú	Native	Bark	Verminoses.	Decoction and maceration; 1 cup 3 x daily for 7 days.	0,04

(80) ***Montrichardia linifera* (Arruda) Schott** (Araceae), IAN 194103	Aninga	Native	Leaves and inflorescences	Healing and general inflammation.	Poultry, topical use 2 x daily for 8 days.	0,09

(81) ***Mora paraensis* Ducke** (Fabaceae), IAN 195702	Pracuúba branca	Native	Bark	Diarrhea, inflammation of the body and irritation of the skin.	Decoction; 1 cup 3 x daily for 7 days.	0,17

(82) ***Musa balbisiana *Colla.** (Musaceae), IAN 194111	Bananeira	Native	Leaves	Hemorrhage and high blood pressure.	Decoction; 1 cup 3 x daily for 7 days.	0,06

(83) ***Ocimum basilicum *L.** (Lamiaceae), IAN 195957	Alfavaquinha	Introduced	Leaves	Cough and catarrh in the chest.	Decoction; 1 cup 3 x daily for 7 days.	0,03

(84) ***Ocimum campechianum *Mill.** (Lamiaceae), IAN 195974	Alfavacão	Introduced	Leaves	Cough and catarrh in the chest.	Syrup and infusion; 1 cup 3 x daily and inhale the steam 2 x daily for 7 days.	0,03

(85) ***Ocimum micranthum *Willd.** (Lamiaceae), IAN 195969	Esturaque	Native	Leaves	Flu, fever and intestinal gas.	Syrup and infusion; 1 cup 3 x daily and inhale the steam 2 x daily for 7 days.	0,25

(86) ***Ocimum selloi *Benth.** (Lamiaceae), IAN 196017	alfavava cheirosa de anis	Native	Leaves and inflorescences	Intestinal gas, inflammation in the stomach, vomiting, flu and fever.	Decoction; 1 cup 3 x daily for 7 days.	0,02

(87) ***Ocotea cymbarum *Kunth** (Lauraceae), IAN 196018	louro-mamorim	Native	Bark	Inflammation in the body and muscle pain.	Decoction; 1 cup 3 x daily for 7 days.	0,04

(88) ***Origanum vulgare *L.** (Lamiaceae), IAN195970	Majerona da angola	Introduced	Leaves and inflorescences	Pains in the body, eliminate phlegm, flu and fever.	Infusion; 1 cup 3 x daily for 8 days.	0,02

(89) ***Otacanthus azureus *(Linden) Ronse** (Plantaginaceae), IAN 195972	Copaibinha	Native	Leaves	Brave wound (leishmaniasis) and inflammation.	Cataplasm, decoction and maceration; 1 cup 2 x daily for 10 days. Topical use 2 x in day for 7 days.	0,62

(90) ***Ouratea hexasperma* (St. Hill.) Benth.** (Ochnaceae), IAN 194112	Barbatimão	Native	Bark	Rheumatism and inflammation in the stomach.	Decoction; 1 cup 3 x daily for 7 days.	0,07

(91) ***Parahancornia amapa* (Huber) Ducke** (Apocynaceae), IAN 196019	Leite do amapá	Native	Exsudate	Infections.	Exudate; 1 cup 2 x daily for 7 days.	0,03

(92) ***Parkia pendula* (Willd.) Benth. ex Walp.** (Fabaceae), IAN 196010	Comadre de azeite- visgo	Native	Bark and inflorescences	Skin wounds and bleeding from the skin.	Decoction and maceration; topical use 2 x daily for 7 days.	0,01

(93) ***Passiflora edulis* Sims** (Passifloraceae), IAN 195936	Maracujá grande	Native	Leaves and bark	Soothing, nervous irritation and inflammation of the stomach.	Decoction; 1 cup 3 x daily for 7 days.	0,24

(94) ***Passiflora tholozanii *Sacco** (Passifloraceae), IAN 196004	Maracujá do mato	Native	Leaves and bark	Brave wound (leishmaniasis), inflammation and soothing.	Decoction; 1 cup 2 x daily for 7 days.	0,02

(95) ***Pedilanthus tithymaloides *Poit.** (Euphorbiaceae), IAN 196021	Coromina, melhoral	Introduced	Bark	Inflammation in the body and verminoses.	Decoction; 1 cup 2 x daily for 10 days.	0,04

(96) ***Pentachletra macroloba *(Willd.) Kuntze** (Fabaceae), IAN 195962	Pracaxi	Native	Leaves, inflorescences and bark	Inflammation in the body and verminoses.	Oil; ingest 1 teaspoon 3x a day for 7 days.	0,83

(97) ***Peperomia pellucida *(L.) Kunth** (Piperaceae), IAN 195961	Erva de jaboti	Native	Leaves	Elimination of kidney stones, cough, sore throats and itchy skin.	Decoction; 1 cup 2 x daily for 10 days.	0,01

(98) ***Petiveria alliaceae *L.** (Phytolaccaceae), IAN 196022	Mucuracaa	Native	Leaves and root	Malaria, pain and phlegm elimination from the chest.	Decoction; 1 cup 2 x daily for 7 days.	0,36

(99) ***Phyllanthus amarus *Schumach. & Thonn.** (Phyllanthaceae), IAN 195955	Quebra pedra	Native	Aerial parts	Urinary tract pain (Urinary tract infection).	Decoction; 1 cup 2 x daily for 7 days.	0,03

(100) ***Physalis angulata* L.** (Solanaceae), IAN 195985	Camapú	Native	Root	Anemia and malária.	Decoction; 1 cup 2 x daily for 7 days.	0,07

(101) ***Platymiscium ulei *Harms** (Fabaceae), IAN 196023	Macacaúba	Native	Bark	Elimination of kidney stones, cough and urinary inflammation.	Decoction; 1 cup 2 x daily for 7 days.	0,01

(102) ***Plectranthus ornatus *Codd.** (Lamiaceae), IAN 194114	Boldo, boldinho	Introduced	Leaves	Flu, acidity and inflammation of the stomach.	Infusion; 1 cup 2 x daily for 7 days.	0,26

(103) ***Pogostemon heyneanus *Benth. **(Lamiaceae), IAN 196024	Oriza	Introduced	Leaves and inflorescences	Flu, phlegm elimination in the chest and cough.	Syrup and infusion; 1 cup 2 x daily for 10 days. Topical use 2 x in day for 7 days.	0,01

(104) ***Portulaca pilosa *L.** (Portulacaceae), IAN 194098	Amor crescico	Introduced	Leaves	Pain in urine (urinary tract infection) and verminoses.	Infusion; 1 cup 2 x daily for 7 days.	0,21

(105) ***Pourouma guianensis *Aubl.** (Urticaceae), IAN 196025	Embaubarana	Native	Bark	Brave wound (leishmaniasis).	Decoction and maceration ingest 1 cup 3 x daily for 7 days Use topic 2 x on day for 7 days.	0,04

(106) ***Pouteria procera *(Mart.) T.D. Penn.** (Sapotaceae), IAN 196026	Maparajuba	Native	Bark	Verminoses and wounds on the skin.	Decoction ingest 1 cup 2 x daily for 7 days.	0,04

(107) ***Psidium guajava *L.** (Myrtaceae), IAN 195950	Goiaba	Native	Leaves	Diarrhea, intestinal and renal infections.	Decoction; 1 cup 2 x daily for 7 days.	0,39

(108) ***Quassia amara *L.** (Simaroubaceae), IAN 195951	Quina	Native	Leaves	Malaria.	Decoction; 1 cup 2 x daily for 7 days.	0,24

(109) ***Ruta graveolens *L.** (Rutaceae), IAN 194106	Arruda	Introduced	Leaves	Ear pain (ear infection), inflammation in the uterus and skin irritations.	Decoction ingest 1 cup 2 x daily for 7 days.	0,23

(110) ***Sambucus australis *Cham. & Schltdl.** (Adoxaceae), IAN 195958	Sabugueiro	Native	Leaves and bark	Measles and chicken pox.	Cataplasm and decoction; 1 cup 2 x daily for 7 days. Topical use 2 x a day for 7 days.	0,02

(111) ***Schinus terebinthifolia *Raddi **(Anacardiaceae), IAN 194105	Aroiera branca	Native	Bark	Healing and inflammation.	Decoction; 1 cup 3 x daily for 7 days. Topical use 2 x a day for 7 days.	0,05

(112) ***Senna reticulata *(Willd.) H.S. Irwin & Barneby** (Fabaceae), IAN 196027	Pacapeá, barajo.	Native	Bark	Elimination of liver stone and rheumatism.	Decoction; 1 cup 3 x daily for 7 days.	0,18

(113) ***Simaba multiflora *A. Juss.** (Simaroubaceae), IAN 196028	Cajurana, Marupaúba.	Native	Bark	Malaria.	Decoction; 1 cup 2 x daily for 7 days.	0,05

(114) ***Siparuna guianensis* Aubl.** (Siparunaceae), IAN 195941	Capitiú	Native	Leaves and bark	Elimination of kidney stones, headache and muscle aches.	Decoction; 1 cup 3 x daily for 7 days.	0,01

(115) ***Spondias mombin *L.** (Anacardiaceae), IAN 195999	Tapereba	Native	Leaves	Inflammation of the mouth and throat and local massages.	Decoction; 1 cup 2 x daily for 7 days.	0,44

(116) ***Stryphnodendron adstringens *Mart.** (Fabaceae), IAN 194113	Barbatimão	Native	Bark	Urinary Tract Pain (Kidney Infections).	Decoction and maceration; 1 cup 3 x daily for 7 days.	0,06

(117) ***Symphonia globulifera* L. f** (Clusiaceae), IAN 194101	Anani	Native	Bark	Anemia and malaria.	Decoction; 1 cup 3 x daily for 7 days.	0,11

(118) ***Syzygium cumini *(L.) Skeels** (Myrtaceae), IAN 194097	Ameixa	Introduced	Bark	Diarrhea and bleeding.	Decoction; 1 cup 3 x daily for 7 days.	0,07

(119) ***Terminalia catappa *L.** (Combretaceae), IAN 195975	Castanhola	Introduced	Bark	Malaria.	Decoction; 1 cup 3 x daily for 7 days.	0,04

(120) ***Theobroma cacau *L.** (Malvaceae), IAN 195967	Cacau	Native	Leaves and seed	Cleaning of body wounds and verminoses.	Decoction and maceration; 1 cup 2 x daily for 7 days.	0,06

(121) ***Theobroma grandiflorum *(Willd. ex Spreng.) K. Schum.** (Malvaceae), IAN 195966	Cupuaçú	Native	Bark and fruit	High and fortifying body pressure.	Decoction; 1 cup 3 x daily for 7 days.	0,03

(122) ***Triplaris surinamensis *Cham.** (Polygonaceae), IAN 195939	Tachí da várzea	Native	Bark	Rheumatism.	Decoction; 1 cup 3 x daily for 7 days.	0,04

(123) ***Uncaria guianensis *(Aubl.) J.F. Gmel. **(Rubiaceae), IAN 196011	Jupindá vermelho	Native	Leaves and bark	Diarrhea, hemorrhage and cancer.	Decoction; 1 cup 3 x daily for 7 days.	0,01

(124) ***Uncaria tomentosa* (Willd.) DC. **(Rubiaceae), IAN 195935	Jupindá	Native	Leaves and bark	Diarrhea, hemorrhage and cancer.	Decoction; 1 cup 3 x daily for 7 days.	0,75

(125) ***Unonopsis floribunda *Diels** (Annonaceae) IAN 195938	Envira-sangue	Native	Bark	Rheumatism and hemorrhage.	Decoction; 1 cup 3 x daily for 7 days.	0,04

(126) ***Vatairea guianensis *Aubl.** (Fabaceae), IAN 196002	Fava de bolacha	Native	Bark and seed	Irritation of the skin.	Decoction; 1 cup 3 x daily for 7 days.	0,05

(127) ***Virola surinamensis* (Rol.) Warb.** (Myristicaceae), IAN 195963	Virola	Native	Bark and fruit	Born in the body (furunculosis) and intestinal infections.	Decoction and exudate as poultice; topical use 2 x a day for 7 days.	0,62

(128) ***Vismia macrophylla *Kunth** (Clusiaceae), IAN 195986	Lacre da folha grande	Native	Bark	Brave wound (leishmaniasis) and skin irritation.	Decoction; 1 cup 2 x daily for 10 days.	0,04

(129) ***Vitex agnus-castus *L.** (Lamiaceae), IAN 195968	Alecrim de angola, Pau de angola	Introduced	Leaves	Cough and phlegm elimination from the chest.	Syrup; 1 teaspoon 3 times a day for 10 days.	0,04

(130) ***Zingiber officinale *Roscoe** (Zingiberaceae), HAMAB 10286	Gengibre	Introduced	Rhizome	Sore throat (inflammation of the throat) and inflammation in the joints.	Decoction; 1 cup 2 x daily for 7 days.	0,01

Native Species: plant that is natural, own of the region of forest of low várzea.

Species Introduced: a species that is established beyond its natural range, after being transported and introduced intentionally or accidentally by man.

**Table 2:** Ethnopharmacological indications of medicinal plants cited by the residents of the river mouth of the Mazagão River.

Category of therapeutic use	Number of plants used	Cited uses	ICF
Microbial Infections	73	532	0,86

Gastrointestinal disorders	47	412	0,89

Inflammatory disorders	41	321	0,88

Pain, fever, cold	25	167	0,86

Malaria	25	90	0,73

Cardiovascular disorders	24	79	0,71

Parasites	23	66	0,66

Respiratory Infections	23	111	0,86

Rheumatism	23	49	0,54

Disorders of the Nervous System	11	45	0,77

Renal, hepatic and biliary disorders	9	30	0,72

Healing	8	41	0,83

Leishmaniasis	8	74	0,9

Cancer	5	58	0,93

Gynecological disorders	5	12	0,64

Metabolic Diseases	3	16	0,87

**Table 3:** Category of therapeutic use and use agreement index (Pcusp) of the medicinal plants cited by the residents of the river mouth of the Mazagão river.

Category of therapeutic use	Scientific Name	NF	FC	Pcusp
Microbial Infections	*Carapa guianensis*	100	1	100
	*Hura crepitans*	100	0,64	64
	*Otacanthus azureus*	96,7	0,68	66
	*Uncaria tomentosa*	97,2	0,82	80
	*Pentaclethra macroloba*	100	0,92	92
	*Copaifera *sp.	100	1	100
	*Dalbergia monetaria*	92,3	0,85	78

Gastrointestinal disorders	*Euterpe oleracea*	89,8	0,62	56
	*Carapa guianensis*	100	1	100
	*Uncaria tomentosa*	100	0,82	82
	*Spondias mombin*	100	0,48	48
	*Dalbergia monetaria*	100	0,85	85
	*Virola surinamensis*	100	0,68	68

Inflammatories disorders	*Carapa guianensis Aubl.*	100	1	100
	*Uncaria tomentosa*	92,1	0,82	76
	*Pentachletra macroloba*	97,5	0,92	90
	*Dalbergia monetaria*	100	0,85	85
	*Virola surinamensis*	79,3	0,54	43
	*Copaifera *sp.	100	1	100

Pain, Fever, Cold	*Plectranthus ornatus*	92,1	0,82	76
	*Eleutherine bulbosa*	97,5	0,92	90
	*Origanum vulgare*	100	0,85	85
	*Petiveria alliacea*	79,3	0,54	43
	*Jatropha curcas*	92,1	0,82	76

Malaria	Symphonia globulifera	100	0,85	85
	Parkia pendula	97,5	0,92	90
	*Petiveria alliacea*	100	0,68	68
	*Maquira coriacea*	92,1	0,82	76
	*Arrabidaea chica*	100	0,85	85
	*Quassia amara*	100	0,68	68
	*Sambucus australis*	92,1	0,82	76

Cardiovascular disorders	*Minquartia guianensis*	92,1	0,82	76
	*Licania macrophylla*	97,5	0,92	90
	*Croton urucurana*	100	0,85	85
	*Arrabidaea chica*	79,3	0,54	43
	*Jatropha curcas*	92,1	0,82	76

Parasites	*Portulaca pilosa*	92,1	0,82	76
	Symphonia globulifera	97,5	0,92	90
	*Cedrela odorata*	100	0,85	85
	*Eleutherine bulbosa*	79,3	0,54	43
	*Arrabidaea chica*	77,1	0,44	34

Respiratory Infections	*Gossypium hirsutum*	92,1	0,82	76
	Adenocalymna alliaceum	97,5	0,92	90
	*Mentha pulegium*	100	0,85	85
	*Eupatorium triplinerve*	79,3	0,54	43
	*Citrus limonum*	96,4	0,32	31
	*Ocimum campechianum*	92,3	0,28	26
	*Chenopodium ambrosioides*	78,6	0,13	10

Rheumatism	*Justicia pectoralis*	60	0,14	8
	*Carapa guianensis*	100	1	100
	*Siparuna guianensis*	92,1	0,82	76
	*Ocotea cymbarum*	97,5	0,92	90
	*Petiveria alliacea*	100	0,85	85
	*Copaifera *sp.	100	1	100

Disorders of the Nervous System	*Aeollanthus suaveolens*	92,1	0,82	76
	*Genipa americana*	97,5	0,92	90
	*Hypericum perforatum*	100	0,85	85
	*Cítrus aurantium*	79,3	0,54	43
	*Passiflora tholozanii*	100	0,02	2
	Passiflora edulis	95,8	0,27	26
	*Eleutherine bulbosa*	70,6	0,14	10

Renal, hepatic and biliary disorders	*Alternanthera brasiliana*	92,1	0,82	76
	*Senna reticulata*	97,5	0,92	90
	*Arrabidaea chica*	100	0,85	85
	*Phyllanthus amarus*	79,3	0,54	43
	*Croton cajucara*	33,3	0,01	0

Healing	*Portulaca pilosa*	92,1	0,82	76
	Montrichardia linifera	97,5	0,92	90
	*Macrolobium acaciifolium*	100	0,85	85
	*Eleutherine bulbosa*	79,3	0,54	43
	*Jatropha curcas*	16,7	0,07	1

Leishmaniasis	*Hura crepitans*	79,6	0,51	41
	*Otacanthus azureus*	96,7	0,68	66
	*Pourouma guianensis*	92,1	0,82	76
	*Gustavia augusta*	97,5	0,92	90
	*Vismia macrophylla*	100	0,85	85
	*Passiflora tholozanii*	79,3	0,54	43
	*Eleutherine bulbosa*	70,6	0,14	10

Cancer	*Alternanthera brasiliana*	52,6	0,12	6
	*Hura crepitans*	94,7	0,64	61
	*Uncaria tomentosa*	97,1	0,8	78
	*Uncaria guianensis*	92,1	0,82	76
	*Maquira coriacea*	97,5	0,92	90
	*Passiflora tholozanii*	100	0,85	85

Gynecological disorders	*Hura crepitans*	79,3	0,54	43
	*Copaifera *sp.	100	1	100
	*Uncaria tomentosa*	97,1	0,8	78
	*Uncaria guianensis*	50	0,01	1
	*Carapa guianensis*	100	1	100
	*Pentachletra macroloba*	97,5	0,92	90
	*Dalbergia monetaria*	100	0,85	85

Metabolic Diseases	*Euterpe oleracea*	81,1	0,51	41
	*Arrabidaea chica*	100	0,85	85
	*Croton cajucara*	79,3	0,54	43

## Data Availability

All data generated and analyzed to support this study are included in this published article. The project was submitted to the Ethics Research Committee of the Faculdade Estácio de Macapá (http://aplicacao.saude.gov.br/plataformabrasil/login.jsf, under the opinion no. 14.94.994). And the complete information of the individual interviews could be requested from the corresponding authors.
